# Epigenetically repressing human cytomegalovirus lytic infection and reactivation from latency in THP-1 model by targeting H3K9 and H3K27 histone demethylases

**DOI:** 10.1371/journal.pone.0175390

**Published:** 2017-04-13

**Authors:** Xin Gan, Haifeng Wang, Yanyan Yu, Wei Yi, Shanshan Zhu, En Li, Yu Liang

**Affiliations:** China Novartis Institutes for Biomedical Research, 4218 JinKe Rd, Pudong, Shanghai, P.R. China; University of San Francisco, UNITED STATES

## Abstract

Human Cytomegalovirus (hCMV) infects a broad range of the population and establishes life-long latency in the infected individuals. Periodically the latently infected virus can reactivate and becomes a significant cause of morbidity and mortality in immunocompromised individuals. In latent infection, the viral genome is suppressed in a heterochromatic state and viral gene transcription is silenced. Upon reactivation, the repressive chromatin is remodeled to an active form, allowing viral lytic gene transcription, initiated by the expression of viral Immediate Early (IE) genes. During this process, a number of histone modification enzymes, including histone demethylases (HDMs), play important roles in driving IE expression, but the mechanisms involved are not fully understood. To get a better understanding of these mechanisms, we focused on two HDMs, KDM4 and KDM6, which reverse the repressive histone H3-lysine 9 and lysine 27 methylation, respectively. Our studies show that in lytic infection, both demethylases are important in the activation of viral IE gene expression. Simultaneous disruption of both via genetic or chemical methods leads to severely impaired viral IE gene expression and viral replication. Additionally, in an experimental latency-reactivation model in THP-1 cells, the KDM6 family member JMJD3 is induced upon viral reactivation and its knockdown resulted in reduced IE gene transcription. These findings suggest pharmacological inhibition of these HDMs may potentially block hCMV lytic infection and reactivation, and control the viral infection associated diseases, which are of significant unmet medical needs.

## Introduction

HCMV is a beta human herpesvirus with a widely spread infection (50% ~ 80% positive adults). It is a major cause of disease and death in immunocompromised patients (HIV-infected persons, organ transplant recipients) and is the most significant viral cause of birth defects in new born infants.[1] However, the efficacy of currently available antivirals is unsatisfactory and vaccines in clinical trials only offer modest protection[2]. The efficacy of current anti-viral drugs (Ganciclovir & derivative) is compromised by the emergence of drug-resistant viruses [3–6] and bone marrow toxicity[7], which limits the usefulness of these compounds in tissue transplant patients. Thus, there is significant unmet need to develop novel anti-virals to control hCMV infection and the associated diseases.

The current anti-hCMV drugs block viral DNA replication, which is a very late stage of viral infection. They do not block expression or functions of viral IE and E gene products, which are immunogenic and can elicit immuno-inflammatory responses that lead to tissue rejection [8] [9] [10]. Additionally, many viral IE gene products are potent transcriptional activators. They may fundamentally alter the status of host cell to favor viral replication and can also serve as an “onco-modulator” to promote oncogenesis in glioblastoma. [11–13] Since the gene transcription of all herpesvirus occurs in a cascade manner (IE to E to L) [1], blocking hCMV IE gene transcription will efficiently halt the viral replication cycle and presumably, prevent the pathogenesis of viral gene products. However, the hCMV major IE promoter (MIEP) contains a variety of elements for transcriptional activation via multiple mechanisms [1, 14], thus it is challenging to repress expression from this promoter. Nevertheless, in recent years, accumulating evidence strongly supports the notion that the assembly and modulation of chromatin associated with the viral genome is an additional layer of complexity in the regulation of viral gene transcription and genome replication. Chromatinisation of hCMV genome occurs in both lytic and latent infection, and the viral gene transcription and genome replication is impacted by histone modification and chromatin modulation.[15–25] These findings suggest that modulation of viral chromatin could represent a new approach to modulate hCMV gene transcription, with the potential to block IE gene expression and shut down viral replication cycle at the initiation stage.

In eukaryotic cells, the cellular genome is packaged into chromatin, which controls many aspects of cellular functions including gene transcription, DNA repair, cellular specification, and development[26]. Chromatin also has a fundamental impact on the gene expression and genome replication of many viral pathogens, especially those double stranded DNA viruses that replicate in the eukaryotic nucleus. These viruses must either utilize chromatin as a support or confront it as an obstacle to complete their life cycles. On the one hand, incoming viral genomes can be the targets of intrinsic cellular defense mechanisms that silence viral gene expression through assembly of repressive chromatin onto the viral genome. On the other hand, viruses can utilize and modulate cellular chromatin-modifying activities to ensure their gene expression and genome replication. [17, 27–31] For a number of herpesviruses, it has been demonstrated that histones with various modifications can be detected associated with viral genomes, various histone modifying enzymes and chromatin modulation proteins are recruited to viral genomes, and altering histone modification or chromatin modulation machinery impacts viral gene transcription and genome replication. [28, 32–34]

Among the histone modifying activities that impact herpesvirus gene transcription, one set of histone demethylases is of particular interest. These enzymes reverse the repressive histone methylation, such as at lysine 9 and 27 of histone H3, [35, 36] thus could potentially be hijacked by these viruses to counteract the transcriptional repression imposed by the host via assembly of repressive chromatin. Therefore, targeting these histone demethylases may compromise the expression of viral lytic genes and potentially block viral replication. Following this strategy, previous studies showed that in Herpes Simplex Virus (HSV-1) models, depleting or chemically repressing two different classes of H3K9 demethylases, LSD1 and the JMJD2 (KDM4) family members impaired viral lytic infection and reactivation from latency. [37–39] To further these studies, using hCMV lytic infection and reactivation models, we examined the role of another HDM, the KDM6 family (members include UTX and JMJD3), which reverses repressive histone H3K27 methylation. Additionally, we tested the potential to enhance the anti-viral effects by combining the inhibition of both KDM4 and KDM6. Our results suggest both KDM4 and KDM6 family members are involved in modulating the repressive viral chromatin and activating viral IE gene transcription. The requirement for these chromatin modulation components opens avenues for the development of novel antivirals that target the initiation of viral gene transcription.

## Results

### CRISPR mediated knockout of JMJD2D (KDM4D) led to reduced HSV and CMV IE gene expression and viral yields

Sharing ~75% amino acid identity within the catalytic JmjC domain, the 4 members (A~ D) of KDM4/JMJD2 family H3K9/K36 HDMs show tissue/cell specific expression and may have distinct target specificities while being biochemically redundant [35, 36, 40, 41]. A recent study [38] demonstrated the JMJD2s are involved to activate HSV and CMV IE transcription in lytic infection. Specific to CMV, JMJD2D knockdown by siRNA appeared to have the most pronounced impact. Thus to directly confirm the role of JMJD2D in CMV IE expression, we constructed knockout cell lines in both 293T cells (HSV surrogate model) and ARPE-19 cells (CMV lytic infection model) using CRISPR technology.

**Fig 1:**
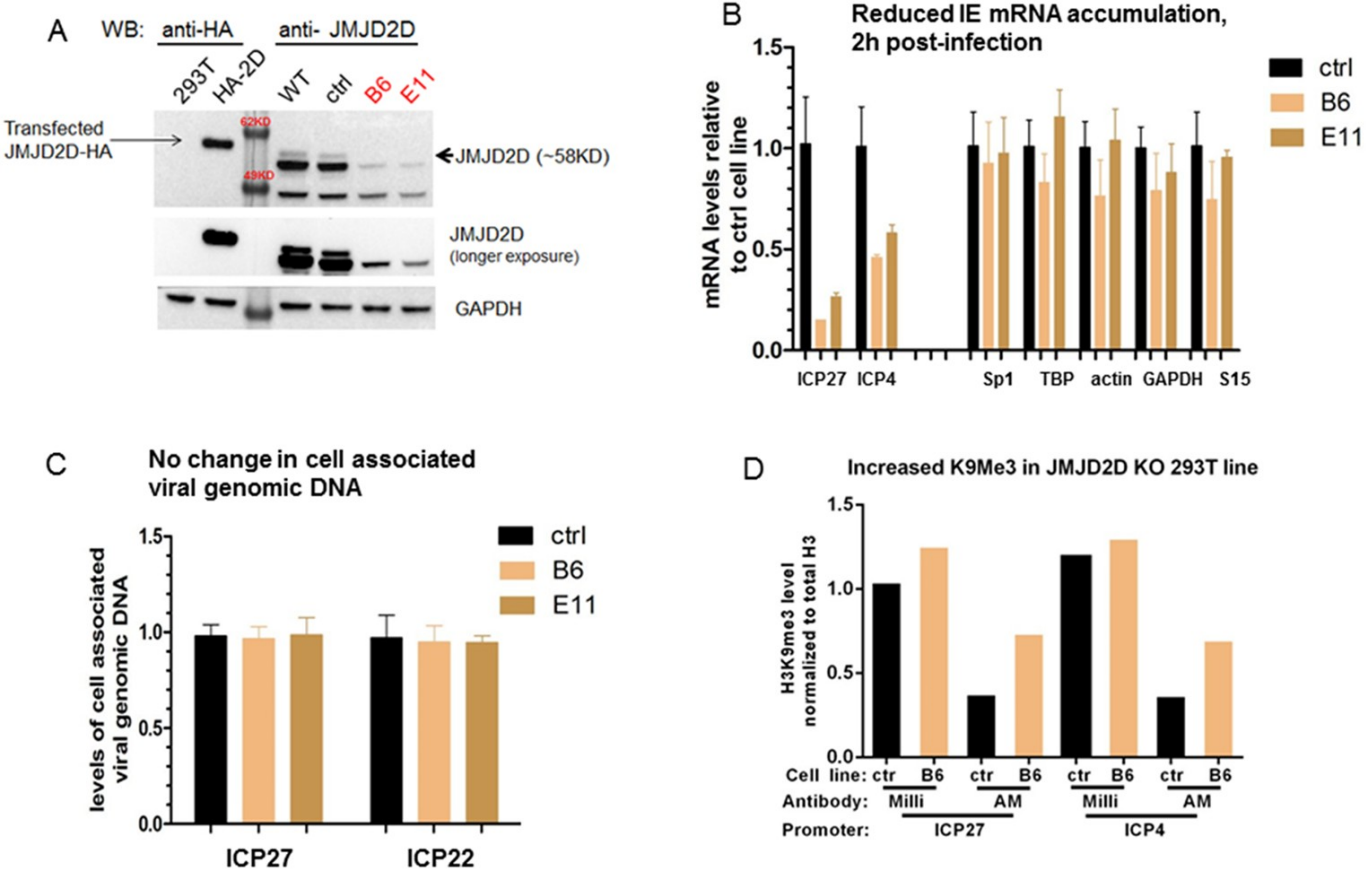
JMJD2D knockout led to reduced IE gene expression in HSV surrogate model. A) Equal amount of cell lysate prepared from mock transfected and JMJD2D-HA transfected 293T, was resolved in the same gel with lysate prepared from 293 WT, control line (derived from control sgRNA transfection) and two JMJD2D KO lines (B6 and E11). The resolved and blotted proteins were probed with anti-HA (1:1000), anti-JMJD2D (1:500), anti-GAPDH (1:1000) respectively. B & C) In duplicate, mock KO (ctrl) or JMJD2D KO 293T lines (B6, E11) were infected with 0.05pfu/cell of HSV for 2hr. Total RNA/DNA was prepared respectively and the levels of viral specific RNA/DNA was determined by qRT-PCR & qPCR, normalized to cellular controls, and expressed as ratios relative to the average of control cell lines. D) H3K9Me3 ChIP assay by two distinct Abs, 2h post infection. Ctrl or JMJD2D KO 293 T cells were infected with 0.1 pfu/cell of HSV-1 for 2hs. The infected cells were cross-linked by formaldehyde, harvested and subjected to chromatin preparation using Millipore EZ-ChIP kit according to the manufacturer’s instructions. Equal amount of chromatin from each cell line was precipitated with anti-H3K9Me3 from two different vendors (Millipore and Active Motif) and anti-total histone H3 (Abcam 1791). The levels of precipitated DNA were quantitated by qPCR with primers against the promoter region of two essential HSV IE genes (ICP27 and ICP4). The H3K9Me3 levels associated with viral IE promoters were normalized to total histone H3 level.

Eight sgRNAs were designed to target the 5’ coding region of the JMJD2D gene to introduce deletion/insertion, leading to frameshift and premature termination within the coding region. After transfection and selection, we obtained two cloned 293T cell lines, B6 and E11, containing the expected insertion/deletion, as confirmed by genomic DNA sequencing (S1A and S1B Fig). To verify the loss of JMJD2D protein in these knockout lines, a western blot was done using equivalent amounts of lysates from the knockout cell lines, the original WT 293T line, and a control line derived in parallel from a mock transfection with a nonspecific sgRNA. As shown in Fig 1A, comparing with the transiently transfected reference with an HA-tag, the full length ~58KD band of JMJD2D was not detectable in both knockout lines (black arrow), suggesting a complete loss of the target protein.

Unlike HSV-1, hCMV does not infect 293T cells. Hence, to evaluate the impact of JMJD2D knockout on herpesvirus IE gene transcription, we first tested HSV-1 infection as a surrogate model, since herpesviruses share many epigenetic mechanisms and components to regulate their gene expression [32–34]. As shown in Fig 1B, at 2 hpi, the transcription of two essential HSV IE genes, ICP4 and ICP27 were reduced in both knockout lines (B6 and E11), compared with the control line. This reduction is not due to the changes in viral infectivity in the knockout lines, since there is no change in cell associated viral genomic DNA after infection (Fig 1C). To determine if the reduced IE gene transcription is associated with elevated histone H3K9 methylation at the viral IE promoters due to the loss of JMJD2D, ChIP assays were performed using two H3K9Me3 antibodies from different vendors. As shown in Fig 1D, a modest increase in the levels of K9Me3 associated with both ICP27 and ICP4 promoters was observed in the knockout line compared with the control line, suggesting the loss of JMJD2D led to a more repressive chromatin conformation. This modest increase is not unexpected, likely due to the existence of other undisrupted JMJD2 family members (JMJD2A/B/C), which were shown to be also involved in HSV IE activation by the previous study. [38]

**Fig 2:**
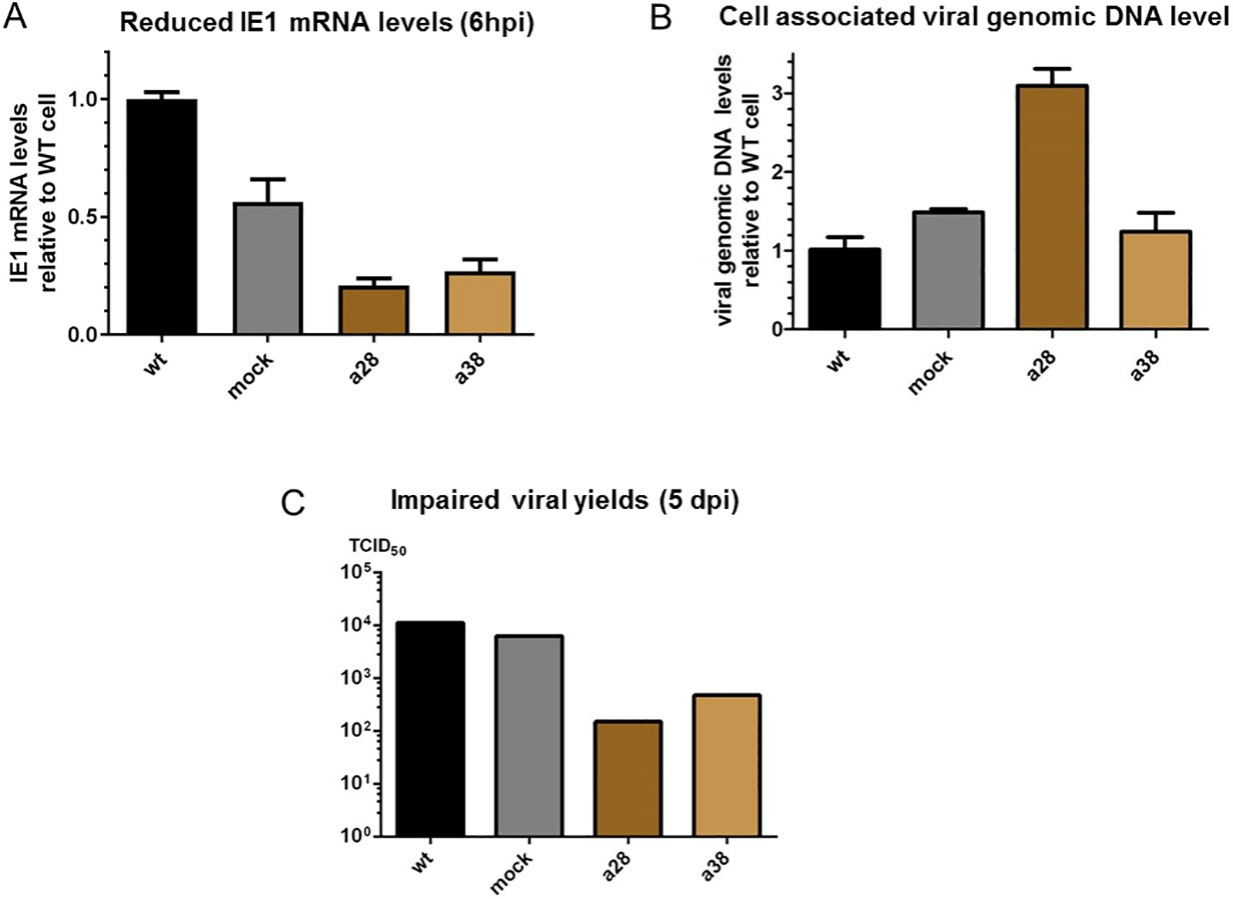
Reduced CMV IE gene expression & viral yields in JMJD2D KO ARPE-19 cells. A &B) In duplicate, ARPE-19 wt, mock KO or JMJD2D KO lines (a28, a38) were infected with same amount of CMV clinical strain (TB40/E) for 6hrs. Total RNA and DNA were prepared respectively and the levels of viral specific DNA/RNA was determined by qPCR/qRT-PCR, normalized to cellular controls, and expressed as ratios relative to the wt cells. To control for the impact on viral IE1 expression level by different multiplicity of infection, the IE1 mRNA level was also normalized to viral DNA level in B). C) The infection described above was extended to 5 days. The total yield of infectious viral progenies (cell associated and supernatant) was measured by TCID50 assay in MRC-5 cells according to standard procedures.

To generate a JMJD2D knockout line which can be infected by hCMV, we utilized ARPE-19 cells, a retinal pigmented epithelium line infectable by some hCMV strains with functional UL131 protein, such as the clinical strain TB40/E[42] or strain AD169 with UL131 gene repaired (AD169-UL131) [43]. Two independently derived CRISPR KO lines were generated (a28 and a38) and confirmed to contain an insertion of “A” at the targeted genomic region by DNA sequencing (S2 Fig). We infected these knockout lines with CMV clinical strain TB40/E and measured viral IE mRNA accumulation (6 hpi), cell associated viral DNA levels at an early stage of infection (6 hpi), and yield of infectious progenies (5 dpi). Compared with the WT line and control line (derived from mock transfection with a nonspecific sgRNA), both JMJD2D knockout lines showed a significant reduction in IE1 mRNA levels (Fig 2A). Additionally, a decreased yield of infectious progenies (1~ 2 log) was observed in JMJD2D knockout lines (Fig 2C). The reduction in viral IE mRNA and viral yields was not due to reduced viral entry of the JMJD2D KO line, because no reduction in the levels of cell associated viral genomic DNA was seen. (Fig 2B).

Taken together, these CRISPR knockout studies suggest JMJD2D plays an important role in stimulating both HSV and hCMV IE gene expression. However, considering the possible functional redundancy of the four JMJD2 family members, solely inhibiting JMJD2D may only be expected to achieve a partial anti-viral effect (~ 50% reduction in hCMV IE expression and 1–2 log less yield of infectious progenies). Thus to efficiently block viral replication, additional targets would need to be identified.

### An H3K27 HDM, JMJD3 (KDM6B), plays important roles in CMV IE gene expression in lytic infection

**Fig 3:**
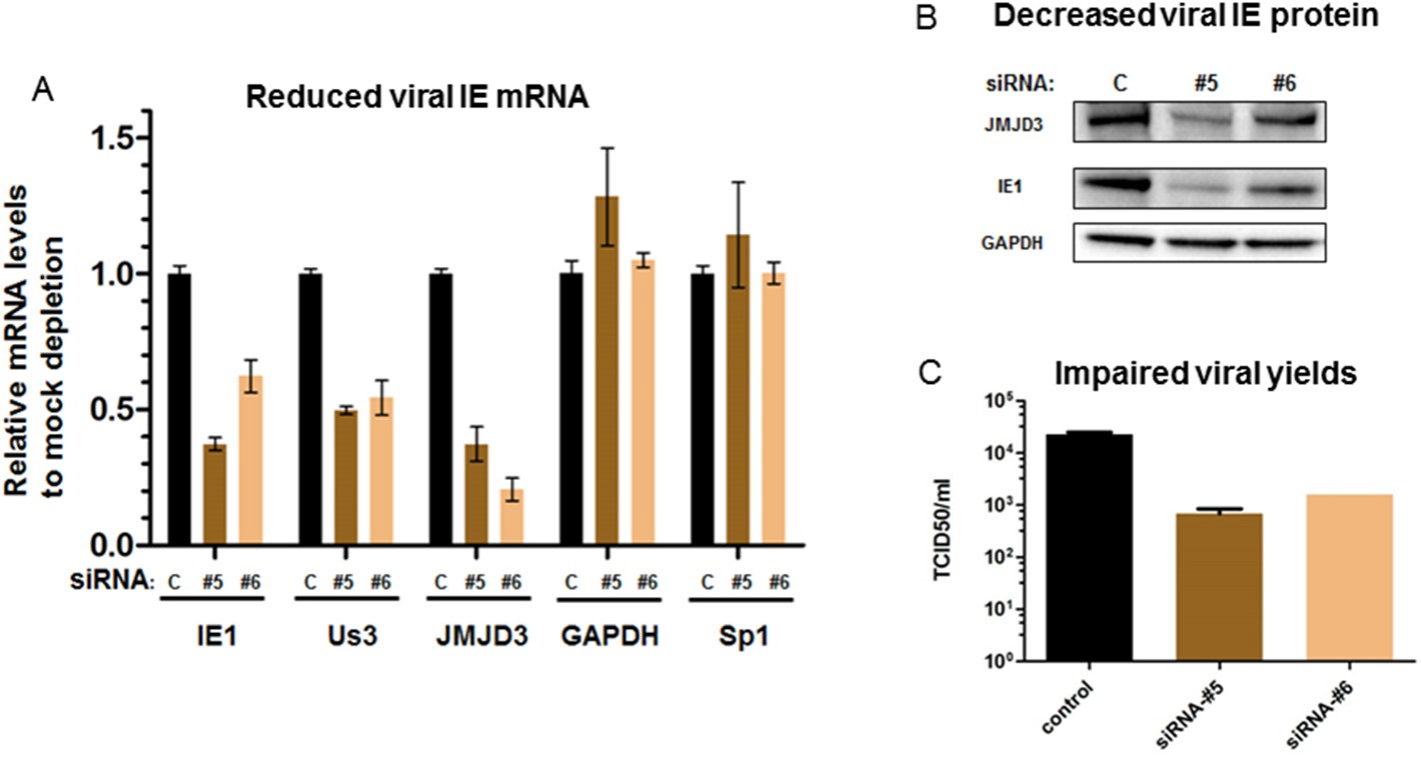
JMJD3 knockdown impaired viral IE gene expression & viral yields in lytic infection. MRC-5 cells were transfected with 20nM control (c) or siRNAs specific to JMJD3 (#5, #6), for 48h; then infected with 0.1pfu/cell of CMV AD169 strain for 6h (for viral mRNA and protein) or 96h (for viral yields). Total RNA was extracted and relevant mRNA levels (A) were quantified by RT-PCR using specific primers. Relevant proteins levels (B) were detected by WB using specific Abs. Viral yields (C) were measured by standard TCID50 assays in MRC-5 cells.

In addition to JMJD2D, the histone H3-lysine 27 (H3K27) demethylase JMJD3, has been implicated in activating IE gene expression of alpha- and gamma- herpesviruses [44, 45].Additionally, the important role of this HDM in the regulation of IE gene expression of the beta-herpesvirus, hCMV, has also been studied. It was shown JMJD3 was recruited to MIEP upon infection to counteract a suppression of IE gene expression by host intrinsic defense mechanisms, such as those mediated by Daxx protein, while viral UL138 protein is involved to resilience the viral genome by preventing JMJD3’s association with the MIEP.[46] Thus, to confirm these studies and better understand the role of JMJD3 in viral lytic infection, we first conducted a series of JMJD3 siRNA knockdown studies. As shown in Fig 3A and 3B, in a MRC-5 lytic infection model, transient transfection with two individual JMJD3 siRNAs led to reduced accumulation of hCMV IE mRNAs (IE1 and Us3) and IE1 protein, but not cellular controls GAPDH and Sp1. As expected, when the yield of infectious progenies was measured by TCID50 assays, JMJD3 knockdown by both siRNAs resulted in ~1 log reduction (Fig 3C). These studies clearly demonstrated that JMJD3 is important for CMV IE expression in lytic infection, thus possibly serving as an additional target to repress viral IE transcription.

### Enhanced repression of viral IE expression and viral yields by combination of JMJD3 knockdown and JMJD2D knockout

**Fig 4:**
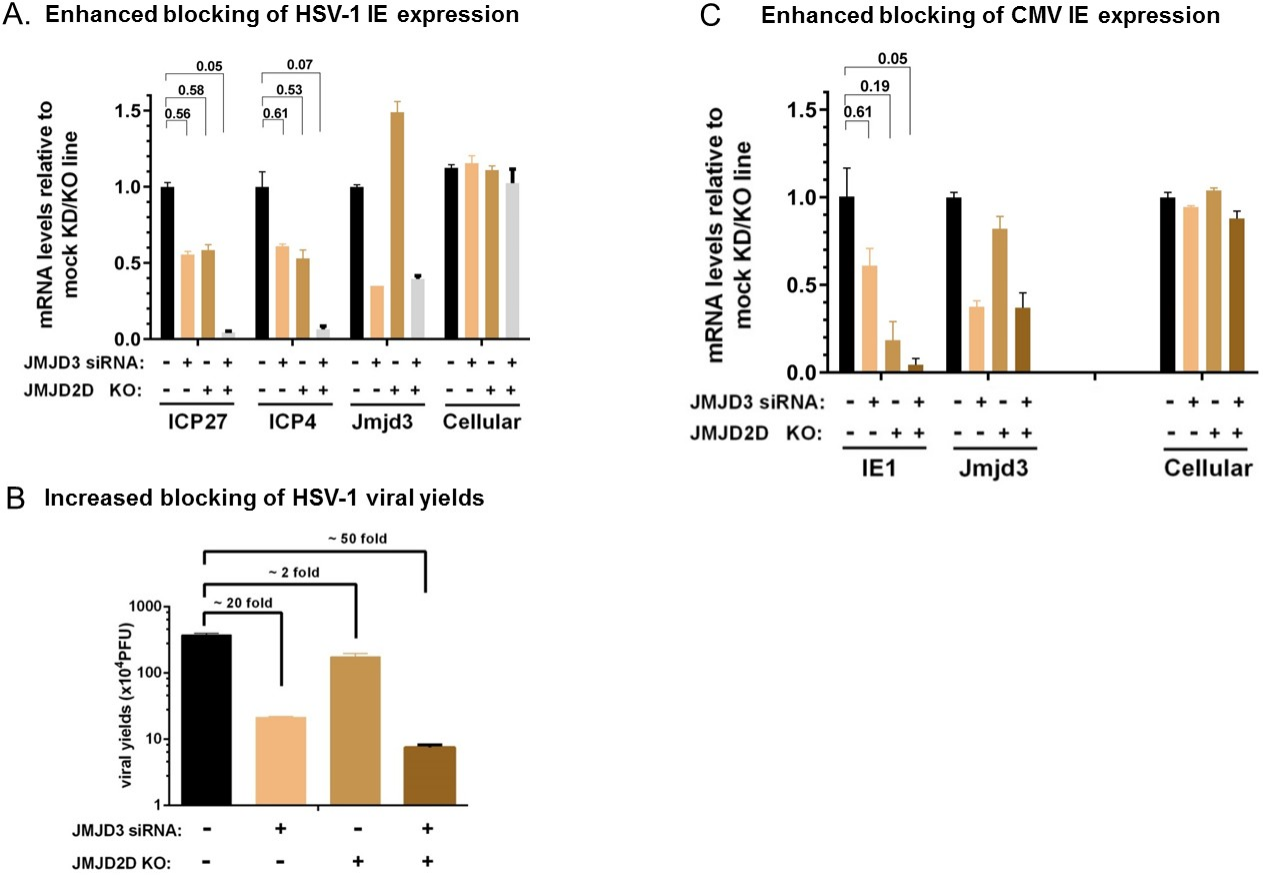
Synergistic repression of viral infection by inhibiting both JMJD2D and JMJD3 upon HSV and CMV lytic infection. A & B) In duplicate, mock KO or JMJD2D KO 293T lines (B6, described previously) were transfected with JMJD3 siRNA#5 or control for 3d, then infected with 0.05pfu/cell of HSV-1 for 2h (for viral IE mRNAs), or 0.005pfu/cell for 24h (for viral yields). Total RNA was prepared and the mRNA levels were measured by qRT-PCR using specific primers and expressed as ratios to mock knockdown/knockout line. The geomean of 4 cellular genes (S15, actin, GAPDH and TBP) was also shown, as “Cellular” control. Viral yields were determined by standard plaque assays in Vero cells. C) ARPE-19 WT or JMJD2D KO clone A28 were mock transfected with control siRNA or JMJD3 siRNA #5, for 3 days, then infected with CMV AD169-UL131 strain for 2hrs. The levels of cell associated viral DNA and mRNAs were quantitated by qPCR or qRT-PCR and normalized to cellular controls. Total RNA was prepared and the mRNA levels were measured by qRT-PCR using specific primers and expressed as ratios to mock knockdown/knockout line. To control for the variation in multiplicity of infection from different cells and its impact on viral mRNA levels, the viral mRNA levels were normalized to the relevant viral DNA level and expressed as ratios to mock depletion in mock knockout line.

We next ask if disrupting both JMJD2D and JMJD3 would lead to a more pronounced repression of viral IE gene expression and replication. For this purpose, we first tried to knock down JMJD3 by siRNA transfection in the JMJD2D knockout 293T line, in an HSV-1 surrogate model. As shown in Fig 4A, respectively knocking down JMJD3 or knocking out JMJD2D reduced HSV IE mRNA (ICP27 and ICP4) by about 50%. However, when both components were disrupted (siJMJD3 in JMJD2D KO cells) a dramatic repression of HSV-1 IE gene expression (~ 95% reduction) was observed, implicating simultaneously inhibiting both HDMs critical for efficient viral IE gene transcription may lead to an enhanced anti-viral effect. As expected, when viral yields are measured, JMJD3 knockdown and JMJD2D knockout led to ~ 20 fold and ~2 fold reduction, respectively; but a more pronounced 50 reduction was observed when they were combined (Fig 4B). Similarly, in a CMV lytic infection model in ARPE-19 cells (shown in Fig 4C), combining JMJD2D knockout with JMJD3 knockdown enhanced the repression of viral IE gene transcription from modest (~ 50% downregulation by KO and KD individually) to very pronounced (>90% repression). These results suggested targeting both histone demethylases that promote viral IE gene transcription resulted in a much more pronounced anti-viral effect.

### An HDM tool compound ML324 inhibits both KDM4 and KDM6 and potently repressed CMV IE gene expression and viral yields

To confirm these results, we used a histone demethylase inhibitor, ML324[47] in the CMV lytic infection model. ML324 is a derivative of the 5-carboxy-8-hydroxyquinoline (IOX1) series, which was first identified in a high throughput screening using JMJD2E and was shown to be an inhibitor of the JMJD2 family HDM [48]. This is confirmed by our quantitative western blot studies measuring global H3K9Me3 levels after compound treatment of MRC-5 fibroblasts (S3A Fig). However, recent studies suggested this IOX1 scaffold likely will have cross-reactivity against other HDMs, especially the KDM6 (JMJD3/UTX) family [49]. To test the *in vivo* substrate specificity of ML324, we employed Mass-spec technology to more comprehensively profile the global acetylation and methylation status of histone H3 after compound treatment. As expected, the results showed (S3B Fig) a pronounced increase in the K9Me2 and K36Me2 level upon ML324 treatment, consistent with its activity as an inhibitor of JMJD2 family (which demethylates both K9 and K36); unexpectedly, we also detected a substantial increase in K27me2, suggesting ML324 also cross inhibits KDM6 family K27 demethylases. Therefore, upon viral infection, treating the host cell with this “dual” HDM inhibitor may simultaneously enhance the levels of repressive histone methylation at both H3K9 and H3K27 associated with viral promoters, thus potentially imposing a “double block” on viral gene transcription, leading to more complete repression of viral replication.

**Fig 5:**
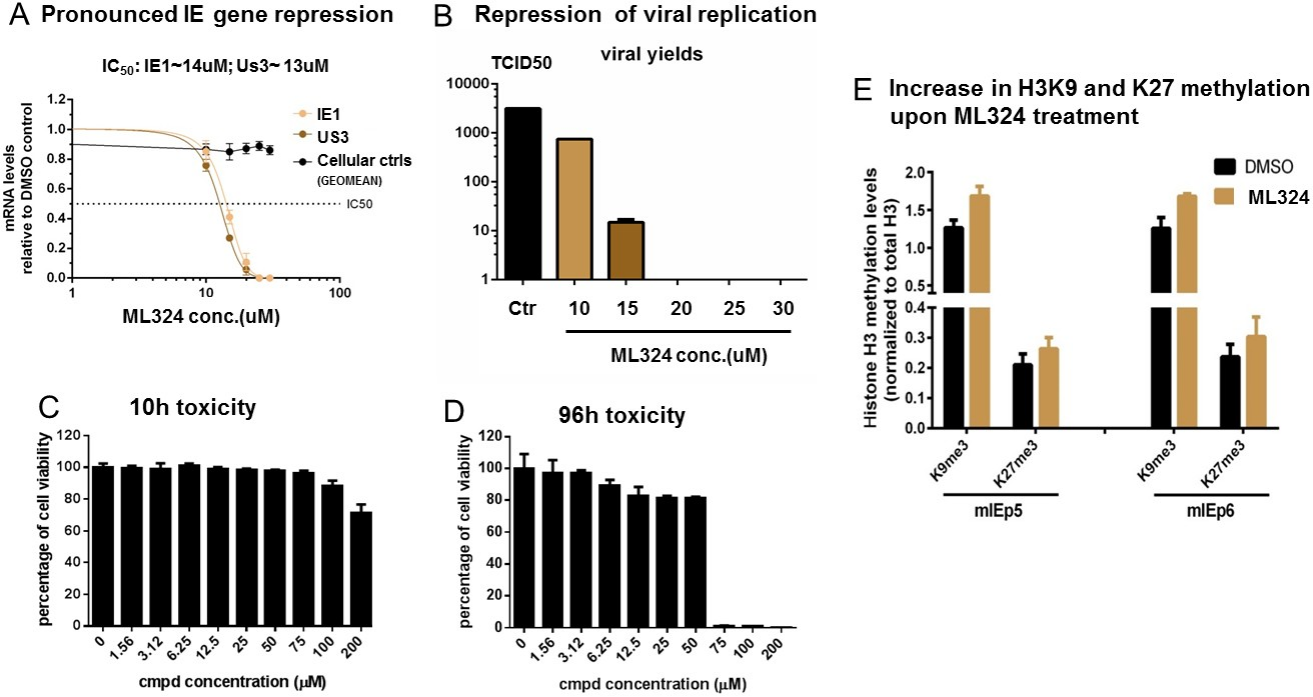
An HDM tool compound ML324 inhibited both KDM4s and KDM6 and potently repressed CMV IE gene expression and viral yields. A & B) MRC-5 cells in duplicate were treated with various concentrations of ML324 or DMSO control for 4h, then infected with 0.1pfu/cell of hCMV AD169 strain, for 5h (to measure mRNA levels), or 96h (to measure viral yields).Total RNA was prepared, reverse-transcribed, and the mRNA levels were determined by qRT-PCR with specific primers of viral IE genes (IE1, Us3), or cellular control genes (GAPDH, Sp1, TBP, S15). The mRNA levels of compound treated samples were expressed as ratios to DMSO control, graphed and analyzed with Prism (version 6.03). Viral yields were determined by standard TCID50 assays. C & D) The cytotoxicity of treatment with various concentrations of ML324 in MRC-5 cells was determined by CellTiter-Glo kit (Promega) after 10h treatment or 96h (corresponding to treating time needed for IE mRNA or viral yield measurement, respectively). E) ARPE-19 cells were mock treated with DMSO or treated with 30uM of ML324 overnight, then infected with 0.1pfu/cell of hCMV AD169-UL131 strain, for 5hr. The levels of K9Me3 and K27Me3 modification associated with viral major IE promoter were determined by ChIP assays using two primer sets (mIEp5 and mIEp6) at different locations within the MIEP, and normalized to total H3 levels. The data shown was compiled from 3 independent experiments and graphed with Prism (version 6.03)

To test this hypothesis, MRC-5 cells were treated with various concentrations of ML324 and infected with CMV for 5hrs (viral mRNA) or 96h (for viral yields). As shown in Fig 5A & 5B, a dosage dependent repression of CMV IE mRNAs (IE1 and Us3, IC_50_ ~ 13uM) and yield of infectious progenies was observed, similar to what is shown in a previous study.[38] Starting from 20uM, ML324 achieved >90% repression of viral IE mRNA and complete block of viral yield. This potent anti-viral effect was unlikely caused by a general toxicity of the compound, since the accompanying cell viability assays showed minimal toxicity at the range of concentrations tested above (Fig 5C & 5D). Next, by chromatin immunoprecipitation assays, we examined the histone H3 methylation level at K9 and K27 associated with the viral major IE promoter (MIEP) upon ML324 treatment. The results showed a mild but consistent increase in both K9Me3 and K27Me3 levels at two separate loci within the MIEP (Fig 5E). This data suggest ML324 treatment may inhibit both K9 and K27 HDMs that are required to reverse the repressive K9 and K27 methylation, thus leading to a more repressive chromatin conformation at viral IE promoters and hence a more pronounced transcriptional repression, compared to individually disrupting either JMJD2D or JMJD3.

### JMJD3 knockdown led to reduced CMV IE gene induction upon reactivation by TPA in THP-1 cells

We next ask if JMJD2D and JMJD3 are important for CMV reactivation, using a well-established CMV experimental latency-reactivation model based on TPA (12-*O*-Tetradecanoylphorbol-13-acetate) induced THP-1 differentiation[50], which recapitulates many key features of CMV latency and reactivation *in vivo*, including the important role of viral chromatin in controlling viral IE gene expression. [51–53] Briefly, we infected undifferentiated THP-1 with CMV AD169 and measured the levels of viral genomic DNA and viral mRNAs daily for 10 days. As shown in S4A Fig, compared with the input level at day 0, viral DNA decreased sharply until day 4, when became stable. Thereafter, viral DNA could be consistently detected until 10 dpi. For viral lytic gene transcription, we observed a rapid decrease in IE1 mRNA until 4 dpi, when it became low (~10% of day 1) but stable. In contrast, for E and L viral mRNAs, although detectable across the time course, these levels never increased substantially. This is in sharp contrast to a viral lytic infection in fibroblast cells, where both viral DNA and mRNAs increase dramatically, in tens of fold, within the first 96 hours after infection. These observations suggested that undifferentiated THP-1 can be infected by CMV, but there is a block to viral IE gene expression and the subsequent viral lytic replication cycle, presumably via the rapid assembly of repressive chromatin onto the invading viral genome, including the major IE promoter [14, 17]. Consequently, the viral genome is quiescently retained in the host THP-1 cell and the lytic gene expression is silenced by repressive chromatin, until reactivated by a number of cellular signaling events, such as those induced by TPA treatment and leading to THP-1 transdifferentiation to macrophage or dendritic cell lineages. Thus as shown in S4B Fig, after 7 days of quiescent infection, TPA can induce THP-1 cells to foster a robust expression of viral IE gene.

Next, with this reactivation model, we examined if any of the well-known epigenetic regulators (as listed in S2 Table) are induced upon TPA treatment and hence might be involved in CMV reactivation. An RNAseq study was performed to compare the transcriptome of TPA induced with mock induced THP-1 cells. The data (S4C Fig) showed JMJD3 was one of the epigenetic genes highly upregulated upon TPA induction. This observation was confirmed by qRT-PCR assays (S4B Fig) showing a substantial upregulation of JMJD3 mRNA at 6hr post induction, concomitant with induction of IE1 mRNA levels.

**Fig 6:**
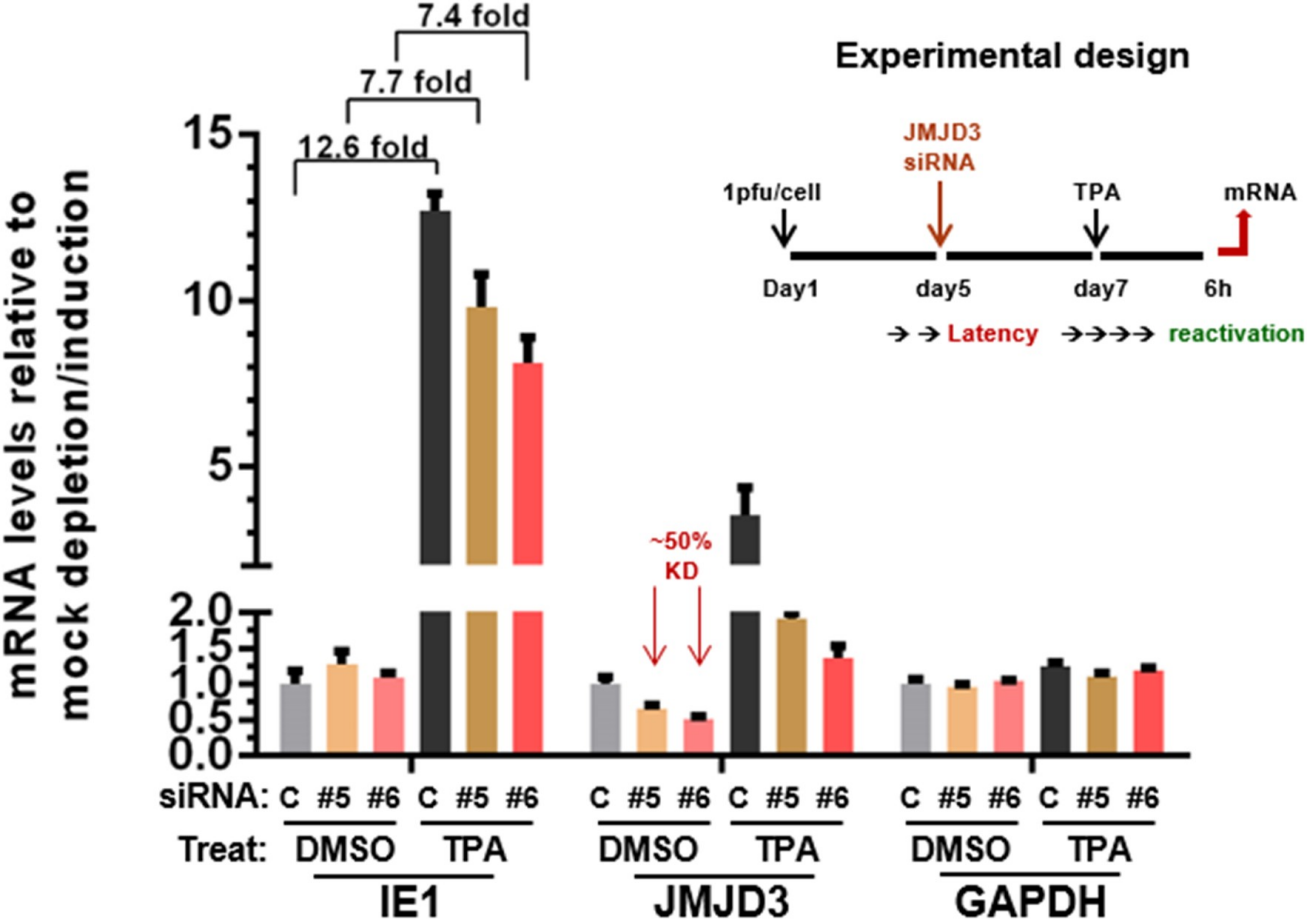
JMJD3 knock-down led to reduced viral IE1 induction upon reactivation by TPA in THP-1 cells. Undifferentiated THP-1 cells were infected with 5pfu/cell of hCMV strain AD169 at day 1, and transfected at day 5 with control or two individual JMJD3 siRNAs (#5 and #6) for 2 days. At day 7, the cells were mock induced or induced with 80nM TPA for 6h. The levels of relevant viral (IE1) or cellular (JMJD3, GAPDH) mRNAs was measured by qRT-PCR with specific primers, and expressed as ratios to mock depleted and mock induced cells.

**Fig 7:**
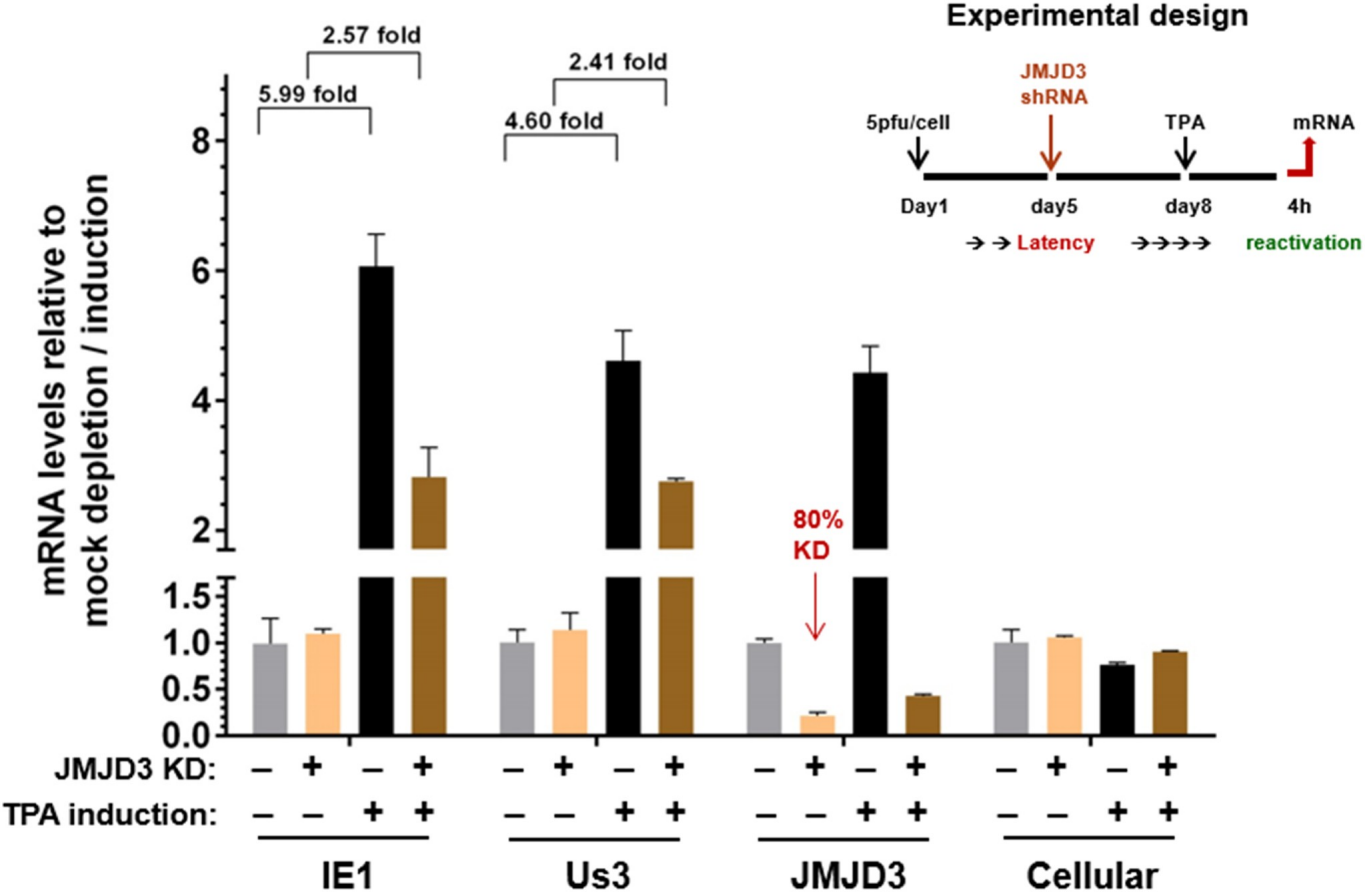
Improved JMJD3 knock-down further reduced CMV IE induction. An undifferentiated THP-1 line inducibly expressing JMJD3 shRNA were infected with 5pfu/cell of hCMV strain AD169 at day 1, and induced at day 5 with doxycycline or mock induced to knockdown JMJD3 expression for 3 days. At day 8, the cells were mock induced or induced with 80nM TPA to reactivate viral gene expression for 4h. The mRNA levels of relevant viral (IE1, Us3), JMJD3 or cellular controls was measured by qRT-PCR with specific primers, and expressed as ratios to mock depleted and mock induced cells.

Thus to further validate the significance of JMJD3 in CMV reactivation, we performed a series of siRNA knockdown studies in the THP-1 reactivation model. As shown in Fig 6, transient transfection with two individual siRNAs led to a ~50% reduction of JMJD3 mRNA. Upon TPA induced reactivation, the fold of IE gene induction was reduced from 12.6 to 7.5 (~40% reduction). To improve the knockdown efficiency, we constructed a THP-1 line inducibly expressing JMJD3 shRNA. As shown in Fig 7, upon doxycycline induction, the efficiency of JMJD3 mRNA knockdown increased from ~ 50% to ~ 80%; and correspondingly, we observed a more pronounced repression of IE gene induction upon TPA treatment. These studies are consistent with an important role for JMJD3 in activation of viral IE expression upon reactivation.

## Discussion

In the current studies using hCMV lytic infection and reactivation models, we focused on the regulatory roles of two sets of HDMs, KDM4 (JMJD2) and KDM6 (UTX/JMJD3), which remove repressive methylation at histone H3K9 and H3K27 respectively. Our results show the following: i) one of the KDM4 members, JMJD2D is involved in activating IE transcription, as its knockout by CRISPR led to reduced IE gene expression and yield of infectious progeny; ii) an H3K27 demethylase, JMJD3 of the KDM6 family, plays an important role in CMV lytic infection, as demonstrated by siRNA knockdown studies; iii) there was enhanced repression of viral IE expression and viral yields by the combination of JMJD3 knockdown and JMJD2D knockout; iv) an HDM tool compound ML324 inhibits both KDM4s and KDM6 and potently repressed viral IE gene expression and viral yields; v) JMJD3 is induced upon hCMV reactivation by TPA treatment in THP-1 cells, and its knockdown led to reduced CMV IE induction. These results suggest both JMJD2D and JMJD3 play important, regulatory roles to activate CMV IE expression and thus, their inhibition may potentially block viral gene expression and genome replication.

In addition to the previously identified H3K9 demethylases (LSD1 and the JMJD2 family)[37–39], the H3K27 demethylase JMJD3, has also been implicated in the induction of CMV IE expression. First, it was shown the H3K27 methyltransferase EZH2 was recruited to the major IE promoter (MIEP) in CD14+ monocytes where hCMV establishes latent infection *in vivo*, resulting in increased H3K27Me3 mark at the MIEP region. [54] Treatment with an EZH2-specific inhibitor reactivates CMV lytic transcription program in THP-1 monocytes, suggesting H3K27 methylation is involved in repression of viral IE transcription during latent infection[55]. In contrast, ectopic expression of the H3K27 demethylases JMJD3 and UTX increased IE mRNA accumulation in latent-infected CD14+ cells [54], suggesting removal of the repressive H3K27 methylation by these demethylases can drive efficient hCMV IE expression and the subsequent reactivation. Secondly, the recruitment of JMJD3 to herpesvirus IE promoters is not unprecedented: it has been demonstrated that JMJD3 can be recruited to the KSHV IE promoter dependent on the viral long noncoding RNA (PAN-RNA)[45], and to hCMV MIEP[46]. However, how JMJD3 is recruited to the hCMV MIEP is still an open question and under further investigation. Lastly, but importantly, in monocytic cells where CMV establishes latent infection, but not in fibroblast or epithelial cells, JMJD3 was induced upon monocyte transdifferentiation into macrophages and dendritic cells, concomitant with viral reactivation.[56, 57] All these observations promoted us to further examine if H3K27 demethylase JMJD3 plays a critical role in hCMV lytic infection and reactivation, and our results strongly suggest this is the case.

Not limited to significant bone marrow toxicity and triggering escape mutants, the current DNA replication inhibitors to treat hCMV infection (i.e. Ganciclovir and derivatives) have a number of other limitations. These compounds do not block the expression of early viral gene products that significantly alter the cellular state and, under some circumstance, are oncogenic. Additionally they do not suppress infection prior to the replication of viral DNA, which results in expression of viral proteins that elicit immune-mediated inflammation and tissue damage− an important component of diseases such as HSV keratitis and hCMV complications in solid organ transplants. Finally, and importantly, they do not efficiently suppress subclinical reactivation and viral shedding, an important mode of viral transmission. Therefore, to address these issues, we tested a different approach: blocking viral IE gene transcription, which is presumably required for viral reactivation, via enhancing the repressive histone modifications (H3K9- and/or K27- methylation) at viral chromatin. Thus the viral reactivation is blocked at the initiation stage and no viral early proteins are produced. This approach may be advantageous with the potential to reduce spontaneous reactivation and related diseases / viral transmission and decrease the inflammatory responses against viral early proteins that mediate additional tissue damage. Actually, in support of this notion, a recent study by Hill *et al*, [58] clearly demonstrated that in a number of HSV *in vivo* reactivation models, epigenetically repressing viral IE gene expression using inhibitors against histone demethylase LSD1 leads to reduced viral primary infection, subclinical shedding, and spontaneous reactivation. Additionally, in another study using HSV-1 mouse model, LSD1 inhibitor tranylcypromine was shown to reduce the *in vivo* viral IE gene expression, decrease the severity of wild type-virus-induced encephalitis (with reduced inflammatory infiltrates) and corneal blindness, block the infection with the acyclovir-resistant HSV-1 mutant, and reduce the *in vivo* viral reactivation in trigeminal ganglia[59]. Taken together, as a proof of principle, to echo with the above mentioned studies in HSV-1 *in vivo* models, the studies presented here provide new evidence supporting the concept of harnessing chromatin control of IE expression as a novel therapeutic approach to control hCMV lytic infection and reactivation, and extend the potential of epigenetic components as drug discovery targets from predominantly the field of oncology [60–62] to viral diseases.[63]

## Materials and methods

### Cells and viruses

MRC-5, THP-1, Vero, ARPE-19 and HEK293T cells were obtained from ATCC and maintained in DMEM or RPMI-1640 medium with 10% FBS according to standard procedures. Viruses were as follows: HSV-1 strain 17 (N. Fraser, University of Pennsylvania), and hCMV strain AD169 (ATCC), TB40/E [42] (gift from Z. Qian, Inst. Of Pasteur, Shanghai), AD169-UL131 (strain AD169 with UL131 gene repaired, gift from N. Jarousse, Novartis Inst. For Biomedical Research, Emeryville, CA).

### Antibodies, Primers, sgRNAs

Antibodies and sequences of the primers and sgRNAs for CRISPR used in these studies are listed in S1 Table.

### Viral yields

Virus from both infected cells and released in the supernant was harvested at 24h (for HSV) or 96h (for hCMV), and tittered using plaque assay in Vero cells (HSV), or TCID50 assay in MRC-5 cells (hCMV), according to standard procedures.

### JMJD2D knockout by CRISPR technology

Briefly, 8 sgRNAs were designed to target the 5’ coding region of JMJD2D gene. Their efficiency of introducing double stranded breaks at the desired loci of the host genome was evaluated by DNA sequencing of the pooled clones after transfection of 293T cells. The two most efficient sgRNAs (#3 and #4) were chosen for follow up. Individual cell clones derived from transfected 293T or ARPE-19 cells with these sgRNAs were further characterized by DNA sequencing. The loss of JMJD2D protein was confirmed by western blot.

### JMJD3 depletion

siRNAs for JMJD3 were obtained from Qiagen, (siRNA #5, Hs_JMJD3_6 SI04133836; siRNA #6, Hs_JMJD3_5 SI03181136) and transfected to target cells using lipofectamine RNAiMAX reagent (Thermo Fisher) according to manufacturer’s instructions. For inducible deletion in THP-1 cells, the targeting sequence of siRNA #6 was inserted to pLKO-Tet-On vector, with which a THP-1 cell line was established after antibiotic selection. The reduced abundance of JMJD3 mRNA after doxycline induction was confirmed by qRT-PCR.

### Western blots

Equal amount of cell lysate was resolved by SDS-PAGE and probed with antibodies listed in S1 Table, according to standard procedures.

### Analysis of viral RNA and DNA

Total DNA from infected cells was isolated using DNeasy Blood & Tissue Kit (Qiagen), quantitated by realtime PCR using specific primers with SYBR green reagent (Applied Biosystems) and ABI Vii7 system, and then normalized to the amount of relevant cellular DNA. Total RNA was prepared using RNeasy Mini Kit (Qiagen), treated with DNase using the DNA-free kit (Ambion) according to the manufacturer’s instructions. Equal amount of total RNA was reverse transcribed using the Maxima First Strand cDNA Synthesis Kit (FMT#K1642), quantitated by realtime PCR using specific primers and normalized to relevant cellular controls.

### ChIP assays

Histone ChIP assays were done essentially as described[64] using the Millipore EZ-ChIP kit(17-371-EZ) according to manufacturer’s instructions. The ChIP data shown is representative of at least two independent experiments.

### Tissue culture based CMV reactivation model

Undifferentiated THP-1 cells were inoculated with 5 pfu/cell of hCMV AD169 strain, transferred to a 6-well plate, centrifuged for 45 min at 700 ×g, followed by 1 h incubation at 37°C. After absorption, the cells were gently washed twice with culture medium, and then incubated for desired time. 80nM TPA was used to induce the transdifferentiation of THP-1 cell and viral reactivation.

### Detection of histone modification by MassSpec

MRC-5 cells in duplicate were treated with HDM inhibitor ML324 or control DMSO for 24h. Histone proteins were prepared by standard acid extraction method, from which histone H3 was purified, derivatized by NHS (Propionate acid N-hydroxysuccinimide ester), digested by trypsin, and subjected to LC-MS analysis using a Sciex Qtrap 5500 mass spectrometer (Applied Biosystems, USA). All data was controlled and synchronized by Analyst software (versions 1.6.1) from AB Sciex. The absolute quantification for each modified peptide was calculated with peak area ratios of target peptide to related inner standard. The relative abundance of each post-translational modification at a certain lysine residue was expressed as percentage of this modification to all modifications examined at the same lysine residue.

### Gene expression analysis by RNA-seq

In duplicates, total RNAs from TPA induced or mock induced THP-1 cells were prepared using miRNeasy mini kit (Qiagen) with on-column DNase I digestion, followed by reversed transcription and sequencing using Illumina platform by Beijing Genomics Institute (BGI). Cleaned RNA-Seq reads from BGI were uniquely mapped to hg19 reference genome using tophat2 with parameters “-g 1 -G knowGene.gtf -m 2”. The genome sequence and known gene annotations for hg19 were downloaded from UCSC genome browser database (http://genome.ucsc.edu/) on 07/26/2014. Expression for each known gene from RefSeq was determined by covered reads and normalized with RPKM (reads per kilobase of exon model per million mapped reads)

## Supporting information

S1 Fig Biallelic KO of JMJD2D by CRISPR in 293T cells. 293T cells were transfected with a construct expressing both JMJD2D sgRNA (#3 or #4) and Cas9 DNA nucleases, and selected for 7 days with antibiotics. Individual clones were picked for preparing genomic DNA. PCR amplified genomic DNA of the gRNA targeted region was subjected to DNA sequencing, to detect indel mutations introduced, after comparing with the wild type clones transfected with a non-specific gRNA. Clone B6 (S1A Fig) contained a biallelic deletion of “C” at the 5’ coding region, resulting a predicted premature stop codon and loss of JMJD2D. Clone E11 (S1B Fig) contained a biallelic insertion of “A” at the 5’ coding region, resulting predicted premature stop codons downstream and loss of JMJD2D protein.
(TIF)

S2 Fig Biallelic KO of JMJD2D by CRISPR in ARPE-19 cells. ARPE-19 cells were transfected with a construct expressing both JMJD2D gRNA #4 and Cas9 DNA nucleases, and selected for 7 days with antibiotics. Individual clones were picked for preparing genomic DNA. PCR amplified genomic DNA of the gRNA targeted region was subjected to DNA sequencing, to detect indel mutations introduced, after comparing with the wild type clones transfected with a non-specific gRNA. Clone A28 and A38 shown here contained a biallelic insertion of “A” at the 5’ coding region, resulting predicted premature stop codons downstream and loss of JMJD2D protein.
(TIF)

S3 Fig Impact of ML324 treatment on global level of H3K9 and H3K27 methylation. A) MRC-5 cells in duplicate were treated with DMSO control or 30μM ML324 for 24h. Total proteins were extracted and detected by western blots using specific Abs to H3K9Me3 modification and total H3. A representative blot of two independent studies was shown. The intensity of each band in A) was measured 5 times by ChemiDoc MP imaging system (Biolab) using different integration/exposure time and their relative levels were calculated using different amount of total cellular proteins on the same blot as a standard. For DMSO and ML324 treatment, the relative level of K9Me3 modification was normalized to total H3 respectively and analyzed by unpaired t-test using Prism (version 6.03), p = 0.0001. **B)** MRC-5 cells in duplicate were treated with the HDM inhibitor ML324 or control DMSO for 24h. Histone H3 protein were purified and subjected to LC-MS analysis. The relative abundance of each post-translational modification at a specific lysine residue was expressed as percentage of this modification to all modifications examined at the same lysine residue. The di-methylation at K9, K27 and K36 increased upon ML324 treatment (comparing red arrows with the blue arrows in the same panel).
(TIF)

S4 Fig Test of the THP-1 based latency-reactivation model. A) Undifferentiated THP-1 cells were infected with CMV AD169 at 5 pfu /cell; the levels of viral genomic DNA and viral mRNAs were examined daily for 10 days by qPCR or qRT-PCR using specific primers, and expressed as ratios to the original level at the start of the studies. B) THP-1 cells in duplicate were infected with 1pfu/cell of CMV for 7 days and induced with 80ng/ml TPA for 6,12 or 24hr. Total RNA was prepared and relevant mRNA levels were determined by qRT-PCR, normalized to cellular controls and expressed as ratios to mock treatment. **C)** In duplicates, total RNAs from TPA induced or mock induced THP-1 cells were prepared, reversed transcribed, and sequenced using Illumina platform by Beijing Genomics Institute (BGI). Cleaned RNA-Seq reads from BGI were uniquely mapped to hg19 reference genome. Expression for each known gene from RefSeq was determined by covered reads and normalized with RPKM (reads per kilobase of exon model per million mapped reads). The genes relevant to epigenetic regulations (154 genes, S2 Table) which showed at least two fold up- or down-regulation were listed in the table.
(TIF)

S1 Table Primers and antibodies. Primer sequences and antibodies used in the study.
(XLSX)

S2 Table Epi-gene list. List of genes involved in epigenetic regulation that were examined in the RNA-seq studies.
(XLSX)
