# Supplementary figures and images for: Epigenetically repressing human cytomegalovirus lytic infection and reactivation from latency in THP-1 model by targeting H3K9 and H3K27 histone demethylases
Source: PLoS One. 2017 Apr 13;12(4):e0175390. doi: 10.1371/journal.pone.0175390 (PMC5391200; doi:10.1371/journal.pone.0175390)

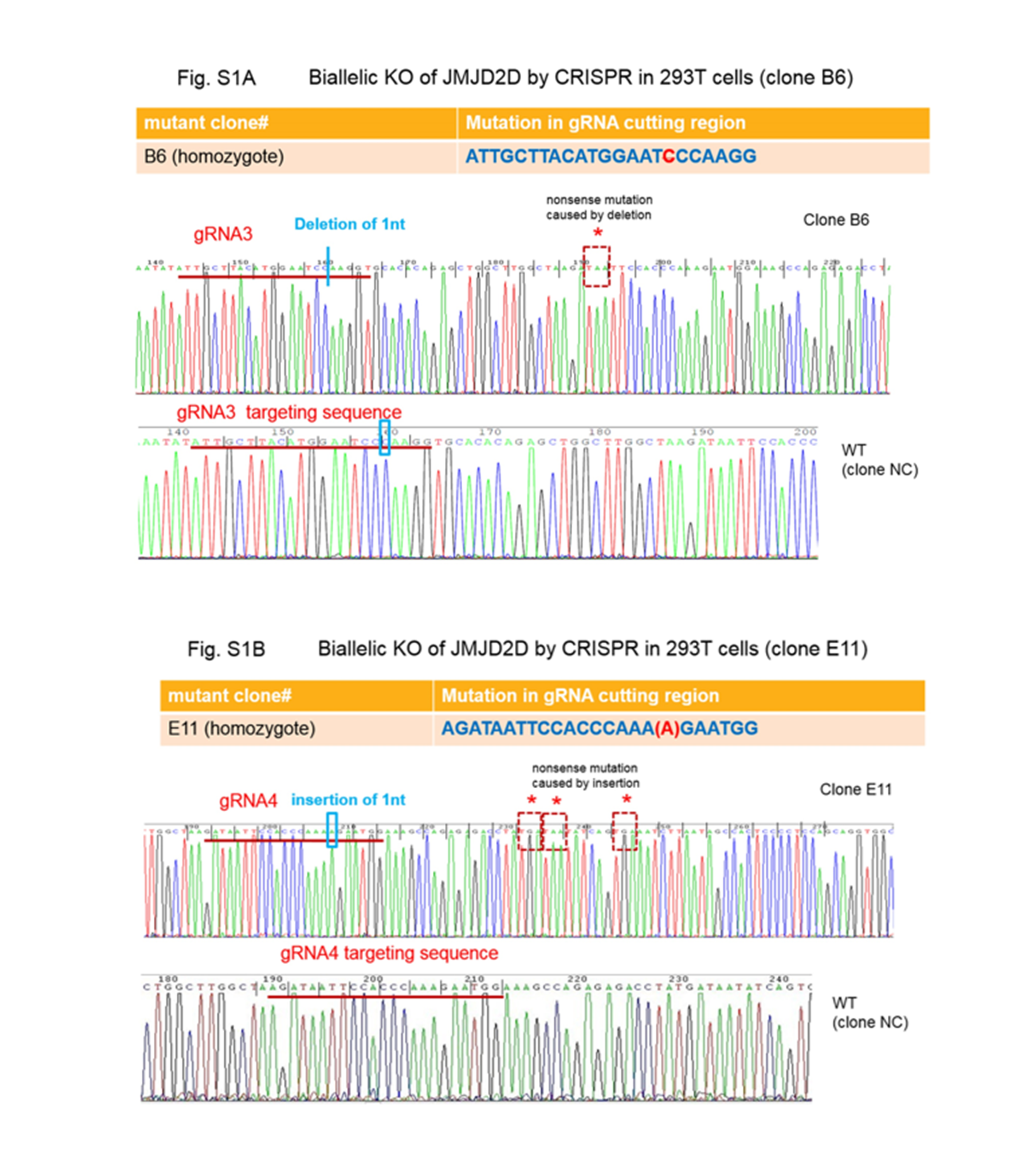

Supplement: S1 Fig — 293T cells were transfected with a construct expressing both JMJD2D sgRNA (#3 or #4) and Cas9 DNA nucleases, and selected for 7 days with antibiotics. Individual clones were picked for preparing genomic DNA. PCR amplified genomic DNA of the gRNA targeted region was subjected to DNA sequencing, to detect indel mutations introduced, after comparing with the wild type clones transfected with a non-specific gRNA. Clone B6 (S1A Fig) contained a biallelic deletion of “C” at the 5’ coding region, resulting a predicted premature stop codon and loss of JMJD2D. Clone E11 (S1B Fig) contained a biallelic insertion of “A” at the 5’ coding region, resulting predicted premature stop codons downstream and loss of JMJD2D protein. (TIF) [file pone.0175390.s001.tif]

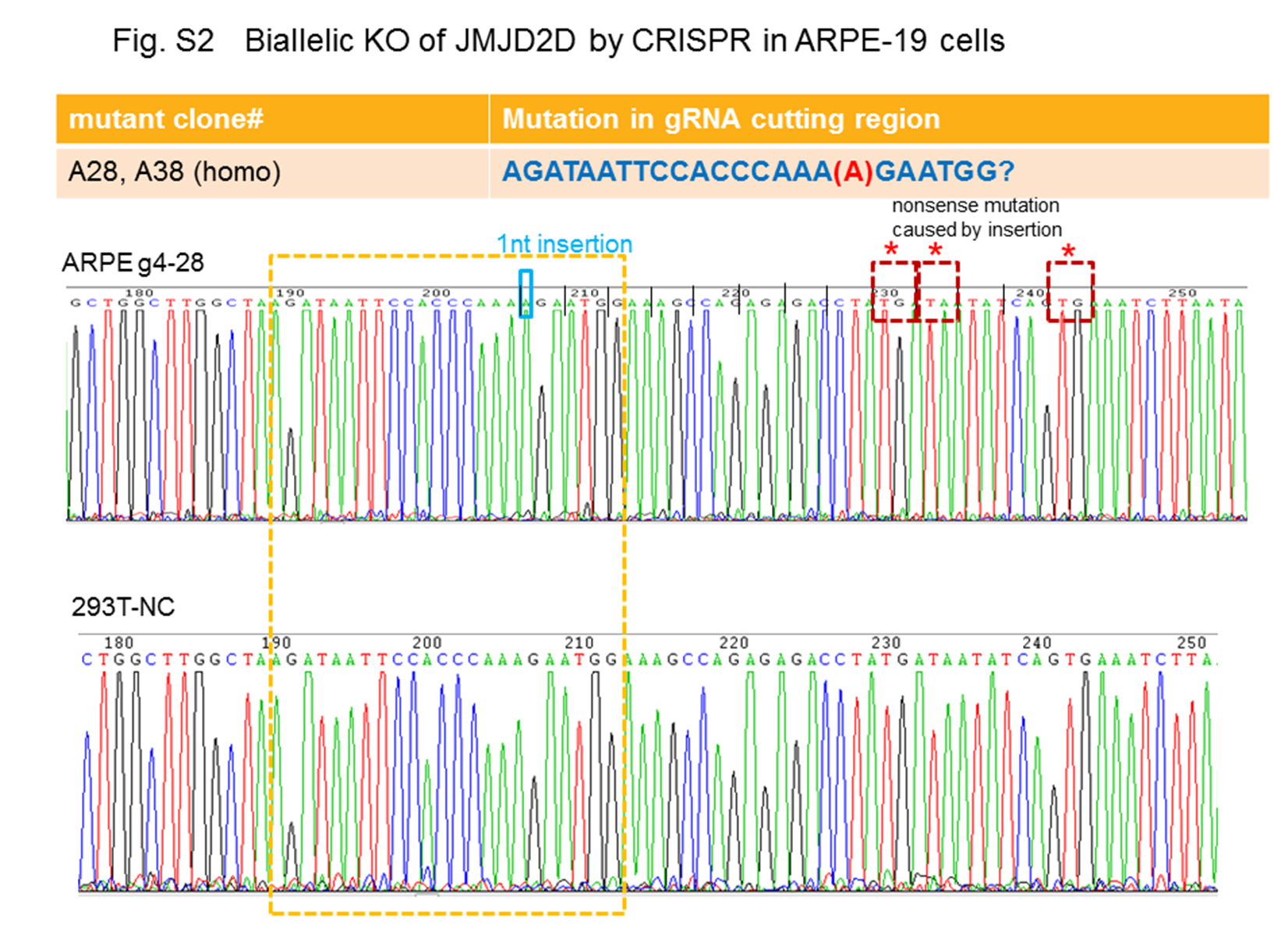

Supplement: S2 Fig — ARPE-19 cells were transfected with a construct expressing both JMJD2D gRNA #4 and Cas9 DNA nucleases, and selected for 7 days with antibiotics. Individual clones were picked for preparing genomic DNA. PCR amplified genomic DNA of the gRNA targeted region was subjected to DNA sequencing, to detect indel mutations introduced, after comparing with the wild type clones transfected with a non-specific gRNA. Clone A28 and A38 shown here contained a biallelic insertion of “A” at the 5’ coding region, resulting predicted premature stop codons downstream and loss of JMJD2D protein. (TIF) [file pone.0175390.s002.tif]

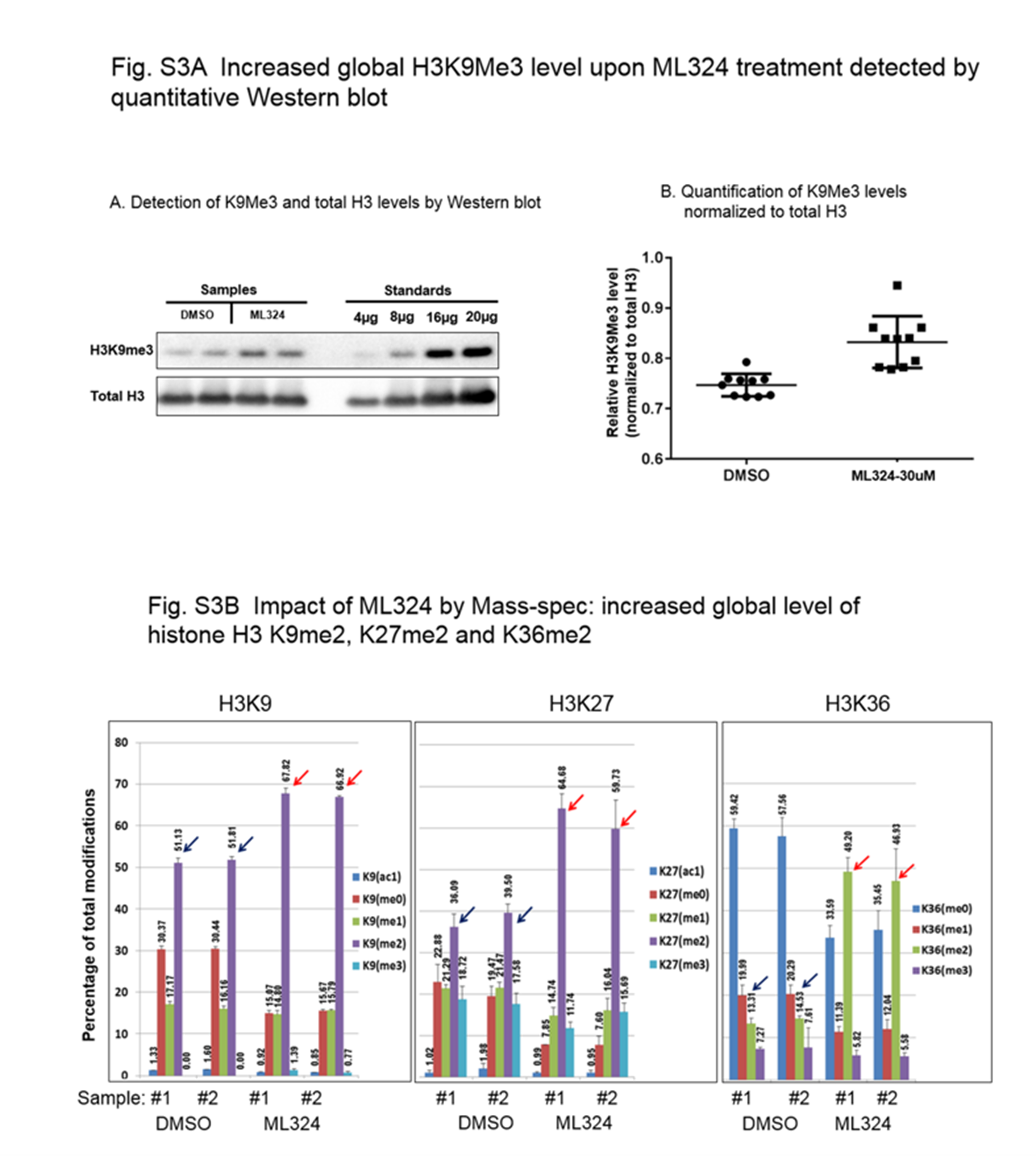

Supplement: S3 Fig — A) MRC-5 cells in duplicate were treated with DMSO control or 30μM ML324 for 24h. Total proteins were extracted and detected by western blots using specific Abs to H3K9Me3 modification and total H3. A representative blot of two independent studies was shown. The intensity of each band in A) was measured 5 times by ChemiDoc MP imaging system (Biolab) using different integration/exposure time and their relative levels were calculated using different amount of total cellular proteins on the same blot as a standard. For DMSO and ML324 treatment, the relative level of K9Me3 modification was normalized to total H3 respectively and analyzed by unpaired t-test using Prism (version 6.03), p = 0.0001. B) MRC-5 cells in duplicate were treated with the HDM inhibitor ML324 or control DMSO for 24h. Histone H3 protein were purified and subjected to LC-MS analysis. The relative abundance of each post-translational modification at a specific lysine residue was expressed as percentage of this modification to all modifications examined at the same lysine residue. The di-methylation at K9, K27 and K36 increased upon ML324 treatment (comparing red arrows with the blue arrows in the same panel). (TIF) [file pone.0175390.s003.tif]

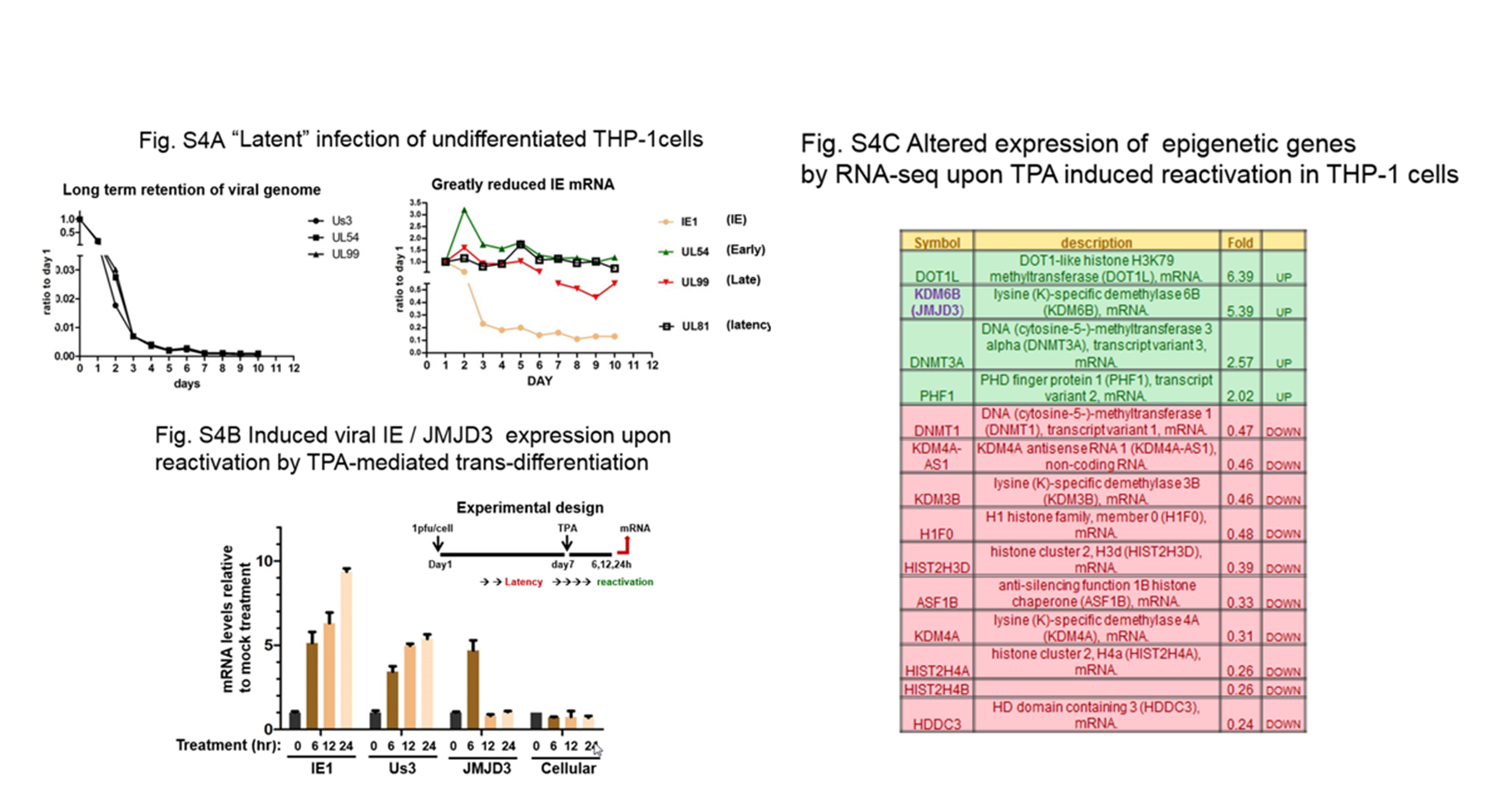

Supplement: S4 Fig — A) Undifferentiated THP-1 cells were infected with CMV AD169 at 5 pfu /cell; the levels of viral genomic DNA and viral mRNAs were examined daily for 10 days by qPCR or qRT-PCR using specific primers, and expressed as ratios to the original level at the start of the studies. B) THP-1 cells in duplicate were infected with 1pfu/cell of CMV for 7 days and induced with 80ng/ml TPA for 6,12 or 24hr. Total RNA was prepared and relevant mRNA levels were determined by qRT-PCR, normalized to cellular controls and expressed as ratios to mock treatment. C) In duplicates, total RNAs from TPA induced or mock induced THP-1 cells were prepared, reversed transcribed, and sequenced using Illumina platform by Beijing Genomics Institute (BGI). Cleaned RNA-Seq reads from BGI were uniquely mapped to hg19 reference genome. Expression for each known gene from RefSeq was determined by covered reads and normalized with RPKM (reads per kilobase of exon model per million mapped reads). The genes relevant to epigenetic regulations (154 genes, S2 Table) which showed at least two fold up- or down-regulation were listed in the table. (TIF) [file pone.0175390.s004.tif]
